# Spatial transcriptomics analysis of zone-dependent hepatic ischemia-reperfusion injury murine model

**DOI:** 10.1038/s42003-023-04564-0

**Published:** 2023-02-18

**Authors:** Jiaqi Xin, Ting Yang, Xiaoyi Wu, Yingting Wu, Yi Liu, Xuan Liu, Mengxi Jiang, Wei Gao

**Affiliations:** 1 School of Traditional Chinese Medicine, Capital Medical University, Beijing, 100069 China; 2 Department of Pharmacology, School of Basic Medical Sciences, Capital Medical University, Beijing, 100069 China; 3 Advanced Innovation Center for Human Brain Protection, Capital Medical University, Beijing, 100069 China; 4 Beijing Shijitan Hospital, Capital Medical University, Beijing, 100038 China

**Keywords:** Liver diseases, Cell biology

## Abstract

Hepatic ischemia-reperfusion (I/R) injury is a common complication in liver transplantation. The connection between I/R-induced injury response and liver heterogeneity has yet to be fully understood. In this study, we converge histopathological examination with spatial transcriptomics to dissect I/R injury patterns and their associated molecular changes, which reveal that the pericentral zones are most sensitive to I/R injury in terms of histology, transcriptomic changes, and cell type dynamics. Bioinformatic analysis of I/R injury-related pathways predicts that celastrol can protect against liver I/R injury by inducing ischemic pre-conditioning, which is experimentally validated. Mechanistically, celastrol likely implements its protective effect against I/R injury by activating HIF1α signaling and represents a potential strategy for resolving liver I/R.

## Abstract (web summary)

Liver zonation across a murine ischemia/reperfusion injury model is characterised and the protective effect of celastrol against I/R injury by activating HIF1α signaling is presented, representing a novel strategy for resolving liver I/R

## Introduction

Hepatic ischemia-reperfusion (I/R) is frequently observed in liver transplantation and hepatectomy with vascular occlusion^1^, which has long affected the success rate of liver transplantation. So far, there has been no approved pharmacological approach for the prevention and treatment of hepatic I/R injury^2,3^.

The liver has a complex, highly compartmented structure and is not an organ that uniformly performs biological functions. The hepatic lobule serves as the basic unit of liver structure and function, which can be divided into the periportal zone (zone 1), the intermediary zone (zone 2), and the pericentral zone (zone 3) according to oxygen supply^4–6^. Different hepatic lobule zones carry out specialized functions^4^. For example, zone 1 hepatocytes have been reported to participate in lipid β-oxidation and gluconeogenesis, while zone 3 hepatocytes are mainly involved in lipid production, ketogenesis, and glycolysis. The transition zone cells are suggested to play an essential role in hepatocyte regeneration^7^. The extent of liver injury in each lobule zone is dependent on the zonation functions. For example, acetaminophen (APAP) is metabolized by zone 3-enriched CYP2E1 and CYP1A2 enzymes, and the resulting toxic metabolites cause pericentral hepatocyte necrosis^8^. In contrast, doxorubicin mainly injures lobule zone 1 due to its preferential metabolism in the more oxidized portal region^9^. Having observed zone-dependent hepatic function and injury pattern, researchers devote to dissecting the liver spatial heterogeneity using techniques such as single-cell sequencing and fluorescence-activated cell sorting^10–12^. In addition to the differential distribution of metabolic enzymes, various factors have been reported to contribute to zone-dependent liver injuries, such as the differences in innate metabolic capacity, oxygen concentration, and adaptive response to ATP crisis between liver zones^12^. Also, hepatic non-parenchymal cells (NPCs) may also contribute to zone-dependent liver injury^11^. Liver I/R has been documented to mainly cause zone 3-specific injury, possibly due to diminished hepatic blood flow during ischemic injury^13,14^. However, spatially resolved molecular dysregulation and cellular dysfunction in liver I/R have not been clarified.

Spatial transcriptomics has revolutionized our understanding of tissue heterogeneity by comprehensive measurement of molecular alterations while maintaining the original tissue spatial context, which offers the possibility to investigate how cell types and tissue heterogeneity cause functional diversity^15^. In the present study, we converged histopathological examination with spatial transcriptomics to dissect pericentral zone-dependent hepatic I/R injury pattern and its associated molecular and cellular changes. We queried the Connectivity MAP (CMap) database^16^ with I/R-regulated and zone-dependent gene signatures, and identified that the small molecule celastrol is most likely to trigger gene perturbations relevant to I/R injury. Celastrol, the main pharmacological component in the Chinese medicinal plant *Tripterygium wilfordii*, has been identified as a potential compound from traditional medicines to be developed into a modern pharmaceutical product^17^. In China and other Asian countries, the root extract of *Tripterygium wilfordii* has long been used to treat chronic inflammatory diseases, such as rheumatoid arthritis^18^. Correspondingly, celastrol is widely reported to reduce inflammation and attenuate ischemia-reperfusion injury in multiple organs^19–21^. Pre-treatment with celastrol reduced liver I/R injury, possibly through activating the HIF1α pathway. Our study comprehensively characterizes liver zonation across I/R injury stages and provides a paradigm for dissecting zone-specific hepatic functions at cellular and molecular levels under physiological and pathological conditions. Celastrol represents a potential strategy for resolving liver I/R.

## Results

### The pericentral zones were most sensitive to hepatic I/R injury in mice

**Fig. 1:**
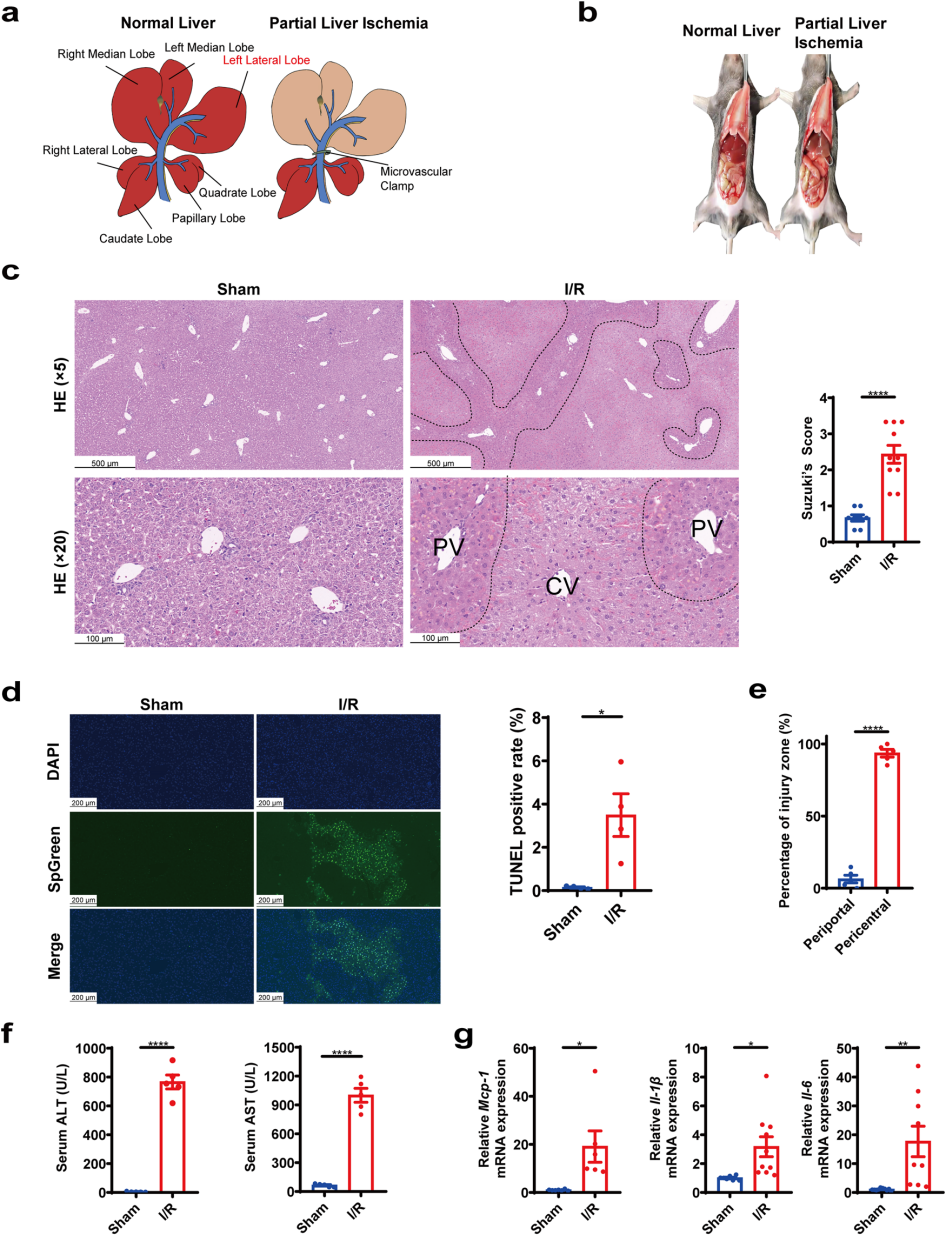
I/R induced zone-dependent injury pattern in mice. **a** Schematic diagram of mouse liver I/R model. Liver morphology (**b**), representative hepatic H&E stainings and histological score (**c**), and representative hepatic TUNEL stainings and TUNEL positivity (**d**) of sham and I/R group. **e** The distribution of I/R injured zones according to H&E stainings. **f** I/R-regulated serum levels of ALT and AST. **g** I/R-regulated hepatic mRNA expression of *Mcp-1*, *Il-1β*, and *Il-6*. Student’s t-test, *n* = 4–10, *p < 0.05, **p < 0.01, ****p < 0.0001. Data were expressed as mean ± SEM. The black dotted line indicates zone-dependent injury region.

Male C57BL/6J mice were subjected to the 70% hepatic I/R model (Fig. 1a). The ischemic liver lobes promptly bleached and turned from reddish to pale brown (Fig. 1b), which indicated the successful establishment of the I/R model. Compared with the sham group, mice in the I/R group demonstrated severe liver injury, showing hepatocellular edema and vacuolation, extensive congestion of liver sinusoids, hepatocyte necrosis, and immune cell infiltration (Fig. 1c). The extent of liver injury of H&E staining was evaluated using Suzuki’s score^22^ (Fig. 1c). The I/R injury also significantly increased the number of TUNEL-positive cells, indicating cellular necrosis and apoptosis (Fig. 1d). Interestingly, the I/R injury pattern was not homogeneous. Rather, the pericentral zone (zone 3) was specifically and most severely injured (Fig. 1e). Serum ALT and AST levels (Fig. 1f) and the mRNA expression of pro-inflammatory cytokines, including *Mcp-1*, *Il-1β*, and *Il-6*, were also induced by I/R injury (Fig. 1g).

### Spatial transcriptomics identified lobule zone-specific genes and functional pathways

**Fig. 2:**
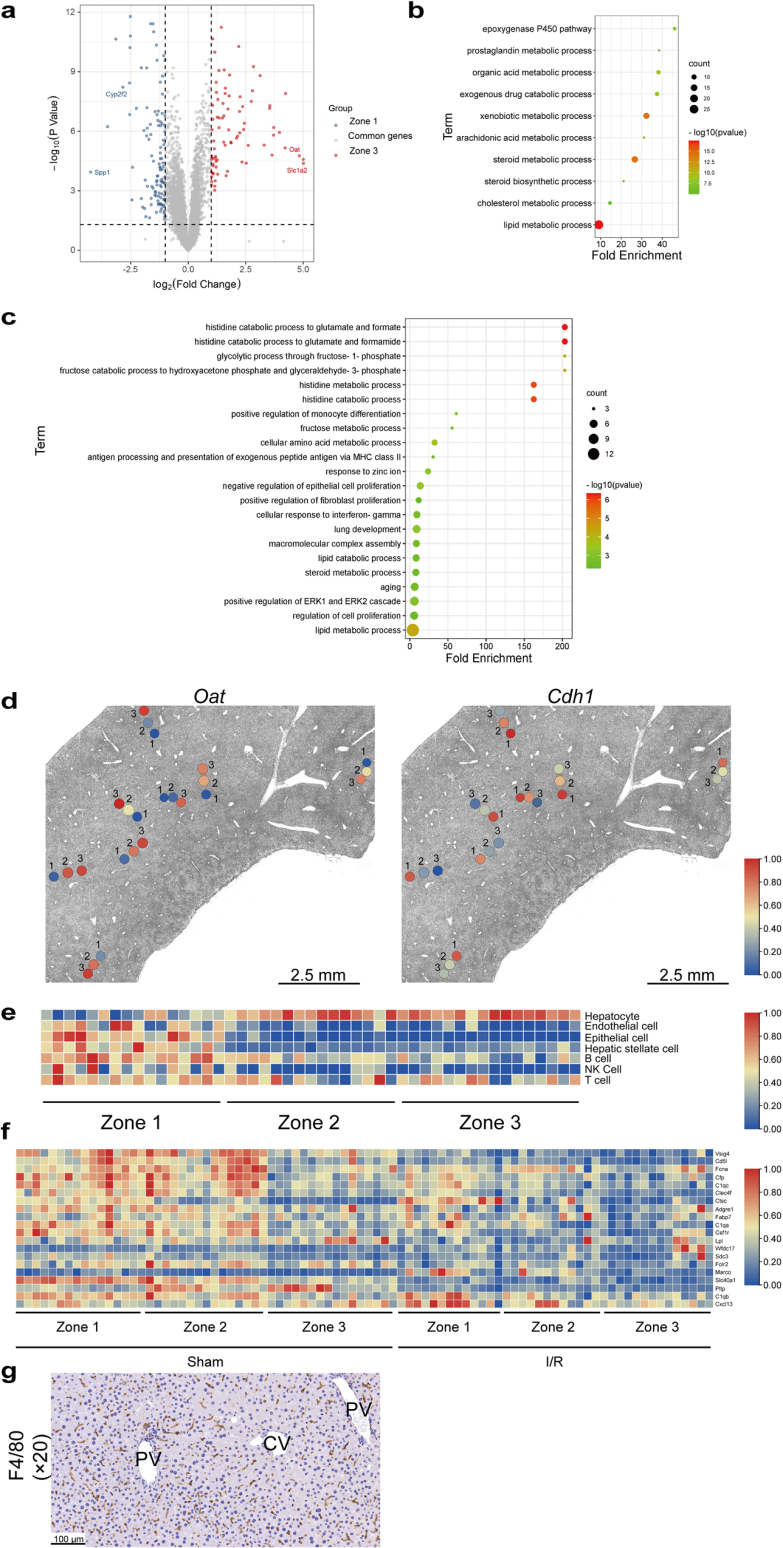
Spatial transcriptomics analysis of lobule zone-specific genes and functional pathways. **a** Hepatic zone-specific DEGs at the steady state. **b** GO analysis of zone 3-specific DEGs. **c** GO analysis of zone 1-specific DEGs. **d** In situ plotting of zone-specific genes in the sham group. **e** Hepatic NPCs and hepatocytes proportion at the steady state. **f** Heatmap of the top 20 KCs marker genes expression. **g** IHC result of Kupffer cell marker F4/80.

Since zonated gene expression patterns contribute to liver heterogeneity and injury response, we used spatial transcriptomics to analyze the zonated gene expression patterns at the steady state (Supplementary Fig. 1a, b). Principal component analysis (PCA) and ROI correlation heatmap were applied to show the repeatability and correlation of each zone (Supplementary Fig. 1d). We identified 191 differentially expressed genes (DEGs) between zone 1 and zone 3 from the sham group (Fig. 2a), including reported zone-specific genes (Fig. 2d, Supplementary Fig. 1e)^7,10^. Through the Gene Ontology (GO) analysis, zone 3-specific DEGs were enriched in endogenous and xenobiotic metabolic pathways (Fig. 2b, Supplementary Fig. 1f), while zone 1-specific DEGs were enriched in histidine and glutamate metabolism and cell proliferation pathways (Fig. 2c), which was consistent with reported zonal functions^23^. To evaluate the spatial distribution of hepatocytes and hepatic non-parenchymal cells (NPCs), cell counts and proportions in each ROI were estimated using the SpatialDecon R package. Enrichment of hepatic NPCs, especially endothelial cells, epithelial cells, and hepatic stellate cells (HSCs) was observed in zone 1 (Fig. 2e). We failed to detect Kupffer cells (KCs) by SpatialDecon, which may be due to the relatively low expression of KCs marker genes. So we evaluated the content of KCs alone according to the absolute expression of their top 20 marker genes and found that KCs were also enriched in zone 1 (Fig. 2f), which was verified by immunohistochemistry (IHC) staining result of Kupffer cell marker F4/80 at steady state (Fig. 2g).

### Spatial transcriptomics analysis of the pericentral zone-dependent I/R injury

**Fig. 3:**
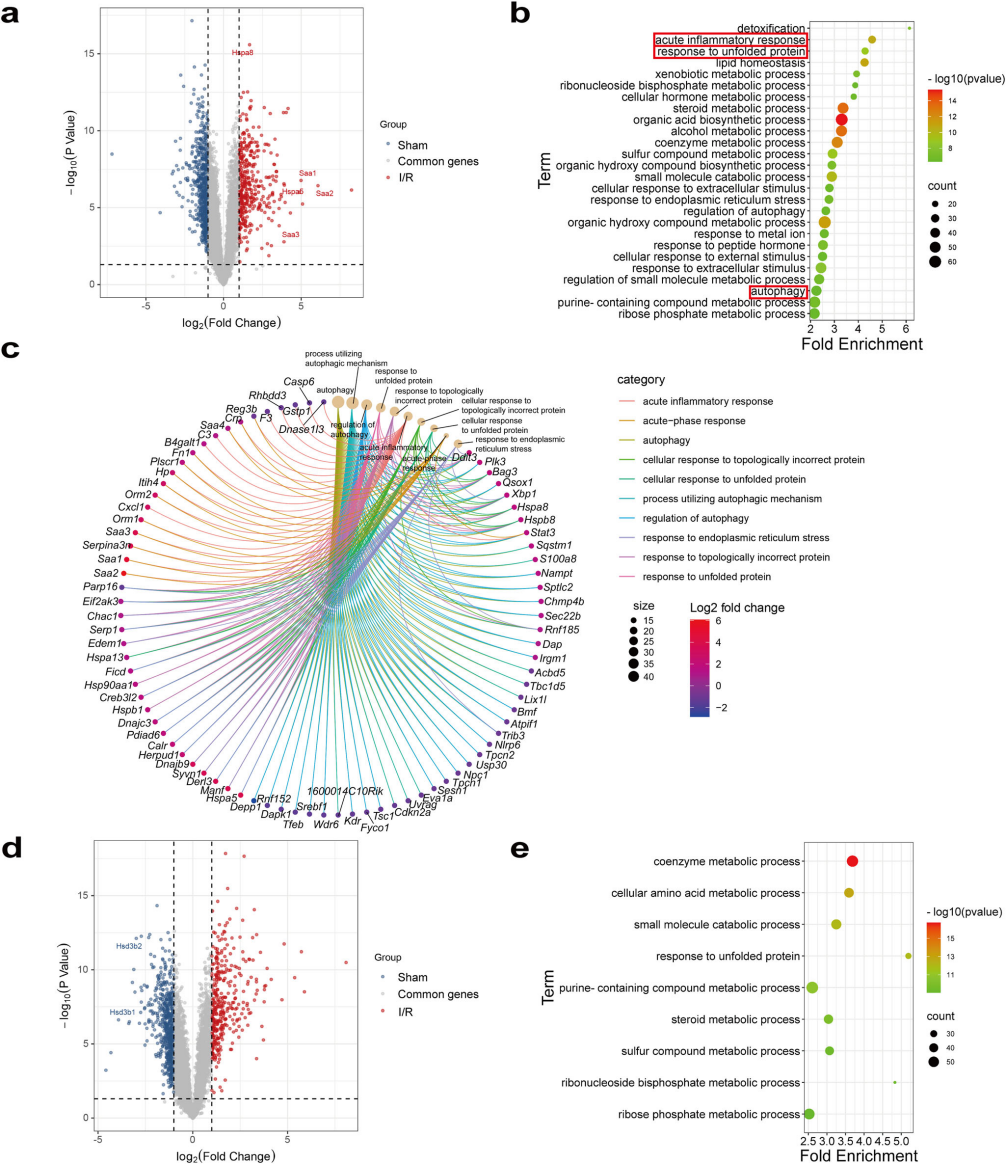
Post-I/R zone-specific DEGs and functional pathways. **a**, **b** Volcano plot and GO analysis of zone 3-specific DEGs of sham and I/R group. **c** Cnet plot of I/R-induced and zone 3-specific DEGs associated with “acute inflammatory response”, “response to unfolded protein”, and “autophagy” pathways. **d**, **e** Volcano plot and GO analysis of zone 1-specific DEGs of sham and I/R group.

**Fig. 4:**
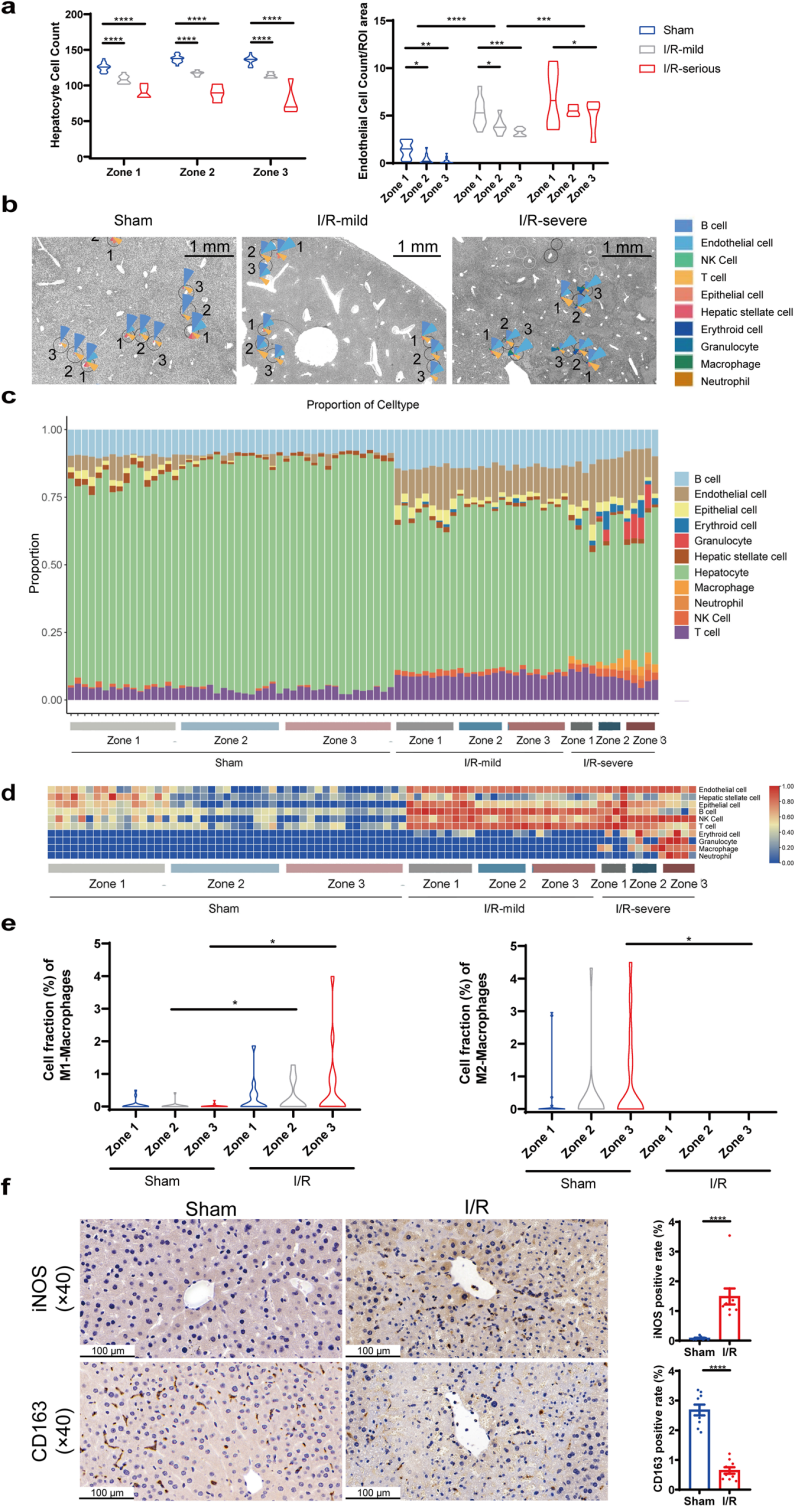
I/R-induced alterations in the proportion and abundance of hepatocytes and hepatic NPCs. **a** The cell count and proportion of hepatocytes and normalized endothelial cells content of sham and I/R groups (two-way ANOVA, *n* = 15–16 for the sham group, *n* = 7–9 for I/R mild regions and *n* = 4–5 for I/R severe regions, *p < 0.05, **p < 0.01, ***p < 0.001, ****p < 0.0001, center lines indicate median value). **b**–**d** Proportion of hepatocytes and hepatic NPCs of sham and I/R groups. **e** Zonal distribution of cell fraction of M1- and M2-macrophages in sham and I/R group (Student’s t-test, *n* = 11–16, *p < 0.05). **f** IHC staining and quantification of M1-macrophages marker iNOS and M2-macrophages marker CD163 (Student’s t-test, *n* = 9, ****p < 0.0001).

To understand the pericentral liver injury pattern in the hepatic I/R model, we analyzed the I/R-regulated DEGs in zone 3 (Fig. 3a) by GO analysis, which were enriched in injury- and metabolic-related functional pathways (Fig. 3b, c) with upregulation of acute phase protein and heat shock protein genes (Fig. 3a, c). While the I/R-regulated DEGs in zone 1 were enriched mainly in metabolic pathways (Fig. 3d, e) with downregulation of steroid metabolism enzymes (Fig. 3d). The spatial distribution and alterations in the content and proportion of hepatocytes and hepatic NPCs following I/R injury were also evaluated. Since the extent of I/R injury is not homogeneous throughout the liver, we assessed changes in hepatic cell populations in mild injury regions and severe injury regions, respectively (Supplementary Fig. 1b, c). I/R injury significantly reduced the number and percentage of hepatocytes (Fig. 4a, c), increased the proportion of endothelial cells and altered the proportion of several other hepatic NPCs (Figs. 2f and 4a–d). Macrophages were mainly recruited to zone 3 of the severe injury regions (Fig. 4b–d). We further calculated the type of infiltrated macrophages by the Cibersort R package and verified the result through IHC, which demonstrated that the content of the pro-inflammatory M1-macrophages and the anti-inflammatory M2-macrophages were induced and reduced, respectively, in zone 3 following I/R injury (Fig. 4e, f), further supporting the zone 3-dependent induction of inflammatory response upon I/R injury. I/R injury also enhanced the proportion of plasma cells and T follicular helper cells (Tfhs) (Supplementary Fig. 2a, b). Therefore, zonated NPCs distribution and content changes may also contribute to zone-dependent hepatic I/R injury response.

### Pre-treatment of celastrol mitigated hepatic I/R injury in mice

**Fig. 5:**
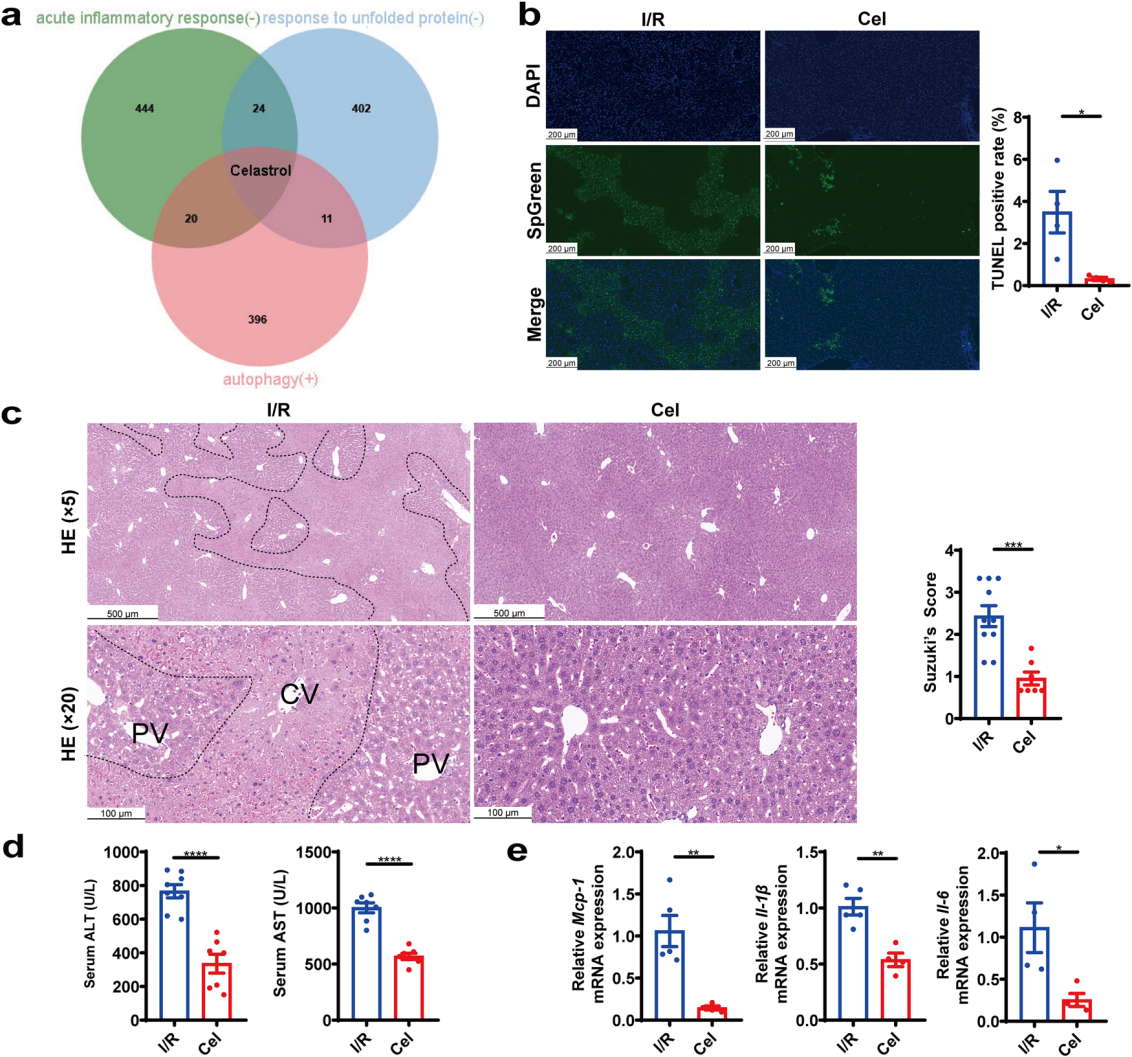
Celastrol (Cel) reduced hepatic I/R injury in mice. **a** The intersection of CMap signatures from I/R regulated and zone 3-specific DEGs involved in UPR, acute inflammatory response and autophagy pathways. **b**, **c** Representative TUNEL staining and TUNEL positivity and representative H&E staining and histological score of I/R and celastrol group. **d**, **e** Serum ALT and AST levels and mRNA expression of hepatic *Mcp-1*, *Il-1β*, and *Il-6* in I/R and celastrol group. Student’s t-test, *n* = 4–10, *p < 0.05, **p < 0.01, ***p < 0.001, ****p < 0.0001. Data were expressed as mean ± SEM. The black dotted line indicates zone-dependent injury region.

In order to identify small molecules that generate similar expression patterns of liver I/R and produce an ischemic pre-conditioning effect that may mitigate hepatic I/R injury, we queried the CMap database with DEGs involved in three of the I/R-regulated pathways in zone 3 (Fig. 3b), namely, response to unfolded protein (UPR), acute inflammatory response and autophagy, whose interventions have been reported to alleviate liver I/R injury^24–26^. The resulting 1500 most relevant signatures were intersected by Venn diagram, with celastrol being the only candidate with strong relevance to the gene expression profile of I/R injury (Fig. 5a). Celastrol has been reported to protect against I/R injury in myocardium, kidney, and nervous system^19–21^. However, the effect of celastrol on hepatic I/R injury has not been reported. In our result, pre-treatment of celastrol alleviated hepatic I/R injury in mice, which was demonstrated by reduced hepatic necrosis and apoptosis from TUNEL and H&E staining results (Fig. 5b, c), reduced serum ALT and AST levels (Fig. 5d and Supplementary Fig. 3a) and decreased mRNA levels of inflammatory genes after hepatic I/R injury (Fig. 5e). Dexamethasone sodium phosphate was applied as a positive control^27^ at the dose of 3 mg/kg, which showed an analogous efficacy as 0.3 mg/kg celastrol at transaminase levels (Supplementary Fig. 3b), suggesting that celastrol exerted a robust protective effect against I/R injury.

### Spatial transcriptomics analysis of celastrol-regulated genes and functional pathways following I/R injury

**Fig. 6:**
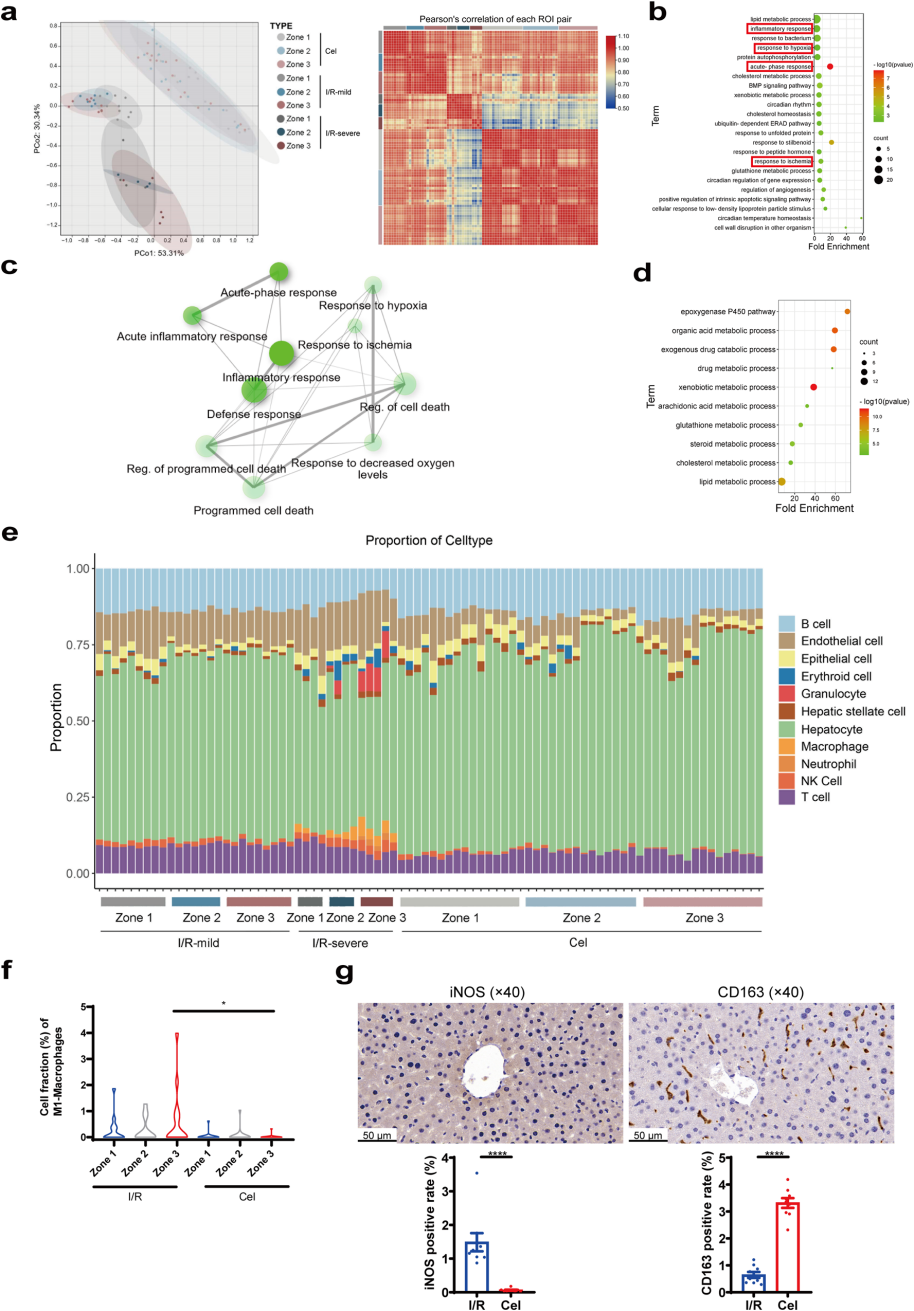
Spatial transcriptomics analysis of celastrol-regulated gene expression and functional pathways. **a** PCA and ROI correlation heatmap of I/R and celastrol group. **b** GO analysis of DEGs of the celastrol group and the I/R group. **c** Network of pathways highlighted in (**b**). **d** GO analysis of zone 3-specific DEGs of the celastrol group. **e** Effect of celastrol on I/R-induced alterations in the proportion of hepatocytes and hepatic NPCs. **f** Cell fraction of M1-macrophages of I/R and celastrol group (Student’s t-test, *n* = 11–16, *p < 0.05). **g** IHC staining and quantification of M1-macrophages marker iNOS and M2-macrophages marker CD163 of celastrol group (Student’s t-test, *n* = 9, ****p < 0.0001).

To identify celastrol-induced gene expression changes after I/R injury, we performed spatial transcriptomics sequencing on 47 ROIs in the celastrol treatment group. PCA well clustered different groups, and the correlation heatmap showed smaller intra-group differences in the celastrol treatment group compared with the I/R group (Fig. 6a). Celastrol-induced DEGs in I/R model were enriched in pathways such as regulation of inflammatory response pathway, and response to hypoxia (Fig. 6b, c). GO analysis showed the gene enrichment patterns in zone 3 of the celastrol group were similar to those of the sham group (Figs. 2b, 6d). Celastrol pre-treatment also blunted I/R-induced macrophage infiltration in zone 3 (Fig. 6e–g) and partially recovered altered NPCs profile induced by I/R injury (Supplementary Fig. 2c, d, f, g).

### The protective effect of celastrol against hepatic I/R injury was likely mediated by HIF1α signaling

**Fig. 7:**
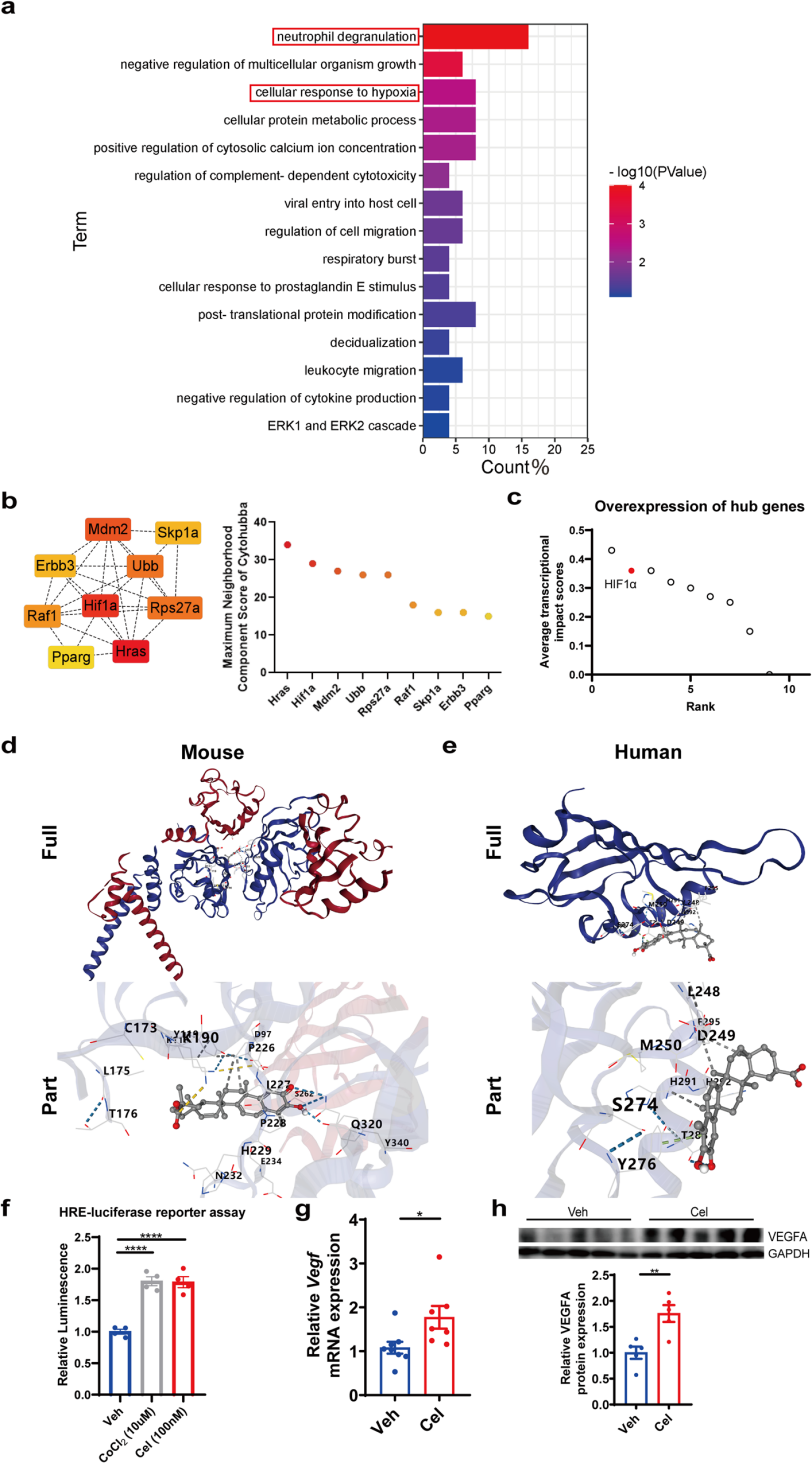
Celastrol activated HIF1α signaling. **a** GO analysis of celastrol-regulated pathways from GSE119291. **b**, **c** The top 9 celastrol-regulated hub genes ranked by Maximum Neighborhood Component scores of Cytoscape and CMap average transcriptional impact scores. **d**, **e** In silico molecular docking results of celastrol with mouse HIF1α protein in (**d**) and human HIF1α protein in (**e**). **f** Effect of 24 h celastrol treatment on transcriptional activity of HIF1α (one-way ANOVA, *n* = 4, ****p < 0.0001). **g**, **h** Celastrol-regulated hepatic mRNA and protein expressions of VEGF (Student’s t-test, *n* = 5–8, *p < 0.05, **p < 0.01). Data were expressed as mean ± SEM.

To understand the function of celastrol at the steady state, we analyzed celastrol-regulated pathways using the GSE119291 dataset^28^, which was enriched in neutrophil degranulation and hypoxia pathways (Fig. 7a). We first selected the top 200 genes from CMap whose overexpression or silencing mimicked celastrol treatment in gene expression regulations. The top 9 hub genes were generated by Cytohubba analysis to identify potential regulons mediating the effect of celastrol, among which HIF1α is upregulated and spatially distributed in the I/R injured mouse liver (Fig. 7b, Supplementary Fig. 2e). HIF1α is also at the forefront of the 9 hub genes according to the CMap average transcriptional impact scores (Fig. 7c). We performed in silico molecular docking of celastrol with mouse and human HIF1α protein and obtained the binding energies of −9.6 kcal/mol and −7.3 kcal/mol (Fig. 7d, e, Supplementary Table 3), respectively. Deferoxamine-mesylate (DFX) was used as a positive control for HIF1α activator in molecular docking simulation and obtained a binding energy of −6.6 kcal/mol between DFX and mouse HIF1α and −4.7 kcal/mol between DFX and human HIF1α (Supplementary Fig. 3c, d, Supplementary Table 3). The lower energy and stable binding between celastrol and HIF1α suggest the possibility of celastrol binding to HIF1α. Celastrol treatment induced the transcriptional activity of HIF1α (Fig. 7f) and the hepatic mRNA and protein expression of *Vegf*, a known Hif1α target gene (Fig. 7g, h and Supplementary Fig. 3e). Therefore, pre-treatment of celastrol likely protected liver I/R injury by stimulating HIF1α signaling and inducing the ischemic pre-conditioning effect.

## Discussion

Liver I/R injury is an urgent problem to be solved in clinical liver transplantation and partial hepatectomy^29^. Though I/R induced zone-specific injury pattern has been observed, the underlying cellular and molecular mechanisms remain unclear. Spatial transcriptomics provided unprecedented opportunities to resolve spatial gene expression patterns and understand spatial tissue heterogeneity^15^, which bridges tissue pathology with gene expression alterations to provide mechanistic insights into liver I/R injury. In the present study, we found that the 70% hepatic I/R model mainly injured the hepatic pericentral zone in mice. Consistent with histology observations, spatial transcriptomics analysis revealed zone 3-specific injury-related DEGs, cellular composition changes and functional pathways following I/R injury. Our study was the first to characterize liver zonation of I/R injury comprehensively and provided a paradigm for dissecting zone-specific hepatic functions at cellular and molecular levels under physiological and pathological conditions.

Recent studies have been applying single-cell sequencing-based techniques to analyze zone-dependent liver injury patterns^11,12,30^. The zone 3-specific damage post APAP overdose was attributed to the lower content of antioxidative glutathione and increased energy requirement in zone 3. Conversely, cells in zone 1 are more resistant to APAP-induced injury due to enhanced mitochondrial function and glutathione content^12^. CCl_4_-induced pericentral fibrosis is mainly due to the pericentral-located HSCs in producing collagen and promoting liver fibrosis^11^. Our study employed spatial transcriptomics to study the spatial organization of hepatic transcriptomic landscape at the steady state and following I/R injury. Histidine and glutamate metabolism pathways are among the top enriched pathways from the zone 1-specific DEGs at the steady state (Fig. 2c), which may improve liver function and alleviate inflammation in liver disease^31,32^ through increasing glutathione-mediated antioxidation effects^23,33^. Meanwhile, the zone 3-enriched lipid metabolism pathways (Fig. 2b) are essential in maintaining hepatic energy and metabolism homeostasis^10^. Hepatocytes apoptosis and necrosis are more likely to occur in the shortage of energy supply due to the more active metabolic functions and energy requirements in pericentral zones. Also, the zone 3-specific injury may be due to low oxygen concentration and deficient expression of hypoxia-related genes in pericentral zones^13,34–36^. Consistently, we observed that compared with zone 1, zone 3 was more sensitive to I/R injury (Fig. 1e). Our study also identified several zone-specific genes (Fig. 2d, Supplementary Fig. 1e). Zone 1-specific *Cyp2f2 and* *Spp1* have been reported to protect the hepatic microenvironment during liver injury^37,38^. While the zone 3-specific *Slc1a2* and *Oat* are likely to reduce glutamate and ornithine content and aggravate I/R injury^39,40^. Therefore, these zone-specific genes not only have the potential to be defined as zone-specific markers, but may also participate in zone-dependent I/R injury.

The complex hepatic cellular architecture is crucial to hepatic I/R injury response. During warm hepatic I/R, hepatocytes are most susceptible to apoptosis and necrosis^41^. Our cell abundance analysis characterized cellular composition before and after I/R injury, which revealed that the percentage of hepatocytes was significantly reduced after warm I/R (Fig. 4a). Spatially heterogeneous function of hepatic NPCs and their abundance in different zones may also contribute to uneven lobule injury through cell-cell interactions and autocrine signaling^42,43^. For example, periportal HSCs and endothelial cells are more proliferative and serve as backup cells that can later migrate into the injury pericentral zone post APAP overdose^30^. In our study, higher expression of endothelial cell marker genes in zone 1 at the steady state indicates higher relative proportion or more active function of endothelial cells (Fig. 2e), which may participate in the protective response against I/R injury possibly through their interactions with monocytes^44^ or activation of Vegfα and its regulated angiogenesis^45^. Zone 1-enriched KCs (Fig. 2f, g) may protect against liver I/R injury through the expression of heme oxygenase-1, NO and glutathione^46,47^. While long-term activation of HSCs drives liver fibrogenesis, short-term activation of zone 1-enriched HSCs, as observed in our I/R model system (Fig. 4b–d), may suppress excessive inflammatory responses and promote liver repair and regeneration after liver I/R injury^48–50^. Hepatic epithelial cells are enriched in lobule zone 1 at the steady state (Fig. 2e), consistent with the location of portal tracts, which contain bile ducts and biliary epithelial cells. The significant increase in endothelial cell proportion after I/R injury (Fig. 4a–d) may also be protective, since endothelial cells were reported to mitigate liver I/R injury and promote liver regeneration^51^. I/R injury has been reported to depress the number and phagocytic activities of KCs^41^. Consistently, we observed declined expression of KCs marker genes after I/R injury (Fig. 2f). Analysis of the macrophage profile and IHC results revealed that the infiltration of the pro-inflammatory M1-macrophages and the anti-inflammatory M2-macrophage were increased and decreased respectively following I/R injury, especially in lobule zone 3, where the injury was more severe (Fig. 4e, f). Plasma cells were reported to regulate protein synthesis pathways and proliferate actively during I/R^52^. Consistently, the proportion of plasma cells was increased following I/R injury (Supplementary Fig. 2a). The increased proportion of Tfhs (Supplementary Fig. 2b) may indicate aggravated I/R injury, since inhibiting the activity of Tfhs was reported to alleviate liver I/R injury^53,54^. Therefore, zonated NPCs patterns may also contribute to zone-specific I/R injury response.

Ischemic pre-conditioning has been demonstrated to confer resistance to subsequent ischemic insult^55^. We discovered through the CMap database that celastrol, one of the main pharmacological components in *Tripterygium wilfordii*, may elicit chemical ischemic pre-conditioning through activating HIF1α, the master regulator of hypoxia pathways. In our study, celastrol was predicted to bind to HIF1α by in silico molecular docking. Celastrol treatment induced the transcriptional activity of HIF1α to the same extent as CoCl_2_ and upregulated the expression of HIF1α target gene (Fig. 7f–h), suggesting that celastrol may serve as an agonist of HIF1α. Activation of HIF1α has been reported to not only confer resistance to hypoxia insult, but also regulate inflammation, autophagy, and UPR, which collectively contribute to HIF1α-mediated hypoxia adaptation^56–58^. Consistently, celastrol treatment activated cellular response to hypoxia pathway (Fig. 7a), and is most likely to trigger gene perturbations relative to autophagy, inflammatory response, and UPR (Fig. 5a) pathways involved in I/R injury, which may serve as the underlying mechanisms of celastrol-induced ischemic pre-conditioning and protection against I/R injury.

In summary, our study contributes to a comprehensive understanding of the spatial heterogeneity of the liver in terms of transcriptomic changes and cell type dynamics across pathological stages, and reveals possible mechanisms of the zone-dependent injury pattern following I/R. Celastrol may elicit chemical ischemic pre-conditioning by activating HIF1α signaling and serve as a potential strategy to prevent hepatic I/R injury.

## Methods

### Drug administration and the mouse model of liver I/R injury

Celastrol was dissolved in saline containing 0.5% DMSO and 0.5% tween 20 (vehicle solution). Male C57BL/6J mice weighing 20–25 g were purchased from HUAFUKANG Bioscience Co., Ltd. (Beijing, China) and housed in the specific pathogen-free (SPF) animal facility of Capital Medical University (Beijing, China) with free access to water and diet. The mice were intraperitoneally injected with either vehicle or different doses of celastrol for 1 week. Mice were fasted for 12 h before being anesthetized with isoflurane and then subjected to 70% liver I/R surgery. A microvascular clamp was placed on the left and middle portal vein branches in the mouse liver to interrupt blood flow to the left and middle liver lobes. Reperfusion was achieved by removing the clamp 1 h after clamping. The mice were sacrificed 6 h after reperfusion, and serum and liver samples were collected for subsequent analysis. All animal experiments were performed under the Guidelines for Animal Care and Use to Medical Research approved by Capital Medical University (AEEI-2021-026).

### Serum chemistry

The mouse serum was collected by centrifugation of clotted blood at 3000 × *g* for 15 min in a refrigerated centrifuge and stored at −80 °C. Commercial kits were used to measure serum levels of ALT (C009-2-1, Jiancheng, Nanjing, China) and AST (C010-2-1, Jiancheng, Nanjing, China) according to the manufacturer’s instructions.

### Liver histology, hematoxylin and eosin (H&E) staining, IHC staining, and terminal deoxynucleotidyl transferase-mediated dUTP nick end labeling (TUNEL) assay

For H&E and TUNEL slides preparation, the left hepatic lobe was fixed with 4% formaldehyde (G1101; Servicebio, Wuhan, China) overnight. Dehydration, wax leaching, deparaffinization, and rehydration were performed with ethanol and xylene. For H&E staining, the slides were stained with hematoxylin and eosin and the histological images of tissue sections were acquired with an upright optical microscope (Nikon Eclipse E100, Japan). Suzuki’s histological score was used to evaluate liver injury according to histological criteria. IHC staining was performed using the following antibodies: Kupffer cell marker F4/80 (1:1000), M1 macrophage marker iNOS (1:500), and M2 macrophage marker CD163 (1:1000). After antigen retrieval, endogenous peroxidase blocking and BSA blocking, primary and secondary antibody incubation and DAB color rendering (G1212; Servicebio, Wuhan, China) were performed. The images were acquired with an upright optical microscope. The quantification of 9 IHC-positive areas from 3 samples was performed by the IHC Profiler plugin of ImageJ software. The TUNEL assay was performed using the FITC TUNEL Assay Kit (G1501; Servicebio, Wuhan, China) following the manufacturer’s instructions. The images were acquired with a fluorescence microscope (Nikon Eclipse C1, Japan). TUNEL-positive cell ratios were calculated by Indica Labs-HighPlex FL v3.1.0 module of Halo software (v3.0.311.314).

### GeoMx DSP Whole Transcriptome Atlas slide preparation

FFPE slides from the left lateral lobe of mice livers were processed following the Manual RNA Slide Preparation Protocol (NanoString, MAN-10115-05 for software v2.3). Briefly, slides were baked at 60 °C for 30 min before deparaffinization, rehydration, and target retrieval. The slides were incubated with 1 μg/mL proteinase K (Ambion, 2546) and the Whole Transcriptome Atlas (WTA) probe reagent at 37 °C in a humidified HybEZ II System for 15 h. The following day, the optimal diluted solutions of morphology markers, including panCK, CD45, and nuclear staining SYTO13 (Nanostring), were used to distinguish cell types and the extent of liver injury.

### ROI selection

The stained tissue slides were scanned with a ×20 microscope for ROI selection using the following parameters: SYTO13 with FITC/525 nm, panCK with Cy3/568 nm, and CD45 with Texas Red/615 nm. The panCK-positive bile ducts of portal triads were used to locate zone 1. H&E and immunofluorescence images were overlaid to localize zone 1 and zone 3. The vascular-free areas between zone 1 and zone 3 were defined as zone 2. H&E staining and CD45-positive immune cell staining patterns were integrated to identify liver injury regions. The areas of vacuolization were defined as mild injury regions, while the areas with prominent necrosis were defined as severe injury regions. Further, CD45-positive rates were calculated as CD45/(CD45 + SYTO13) by ImageJ software, which quantitatively distinguishes the mild- and severe-injured groups by 13% CD45 positivity (Supplementary Fig. 1c). The adjacent zones 1, 2, and 3 are randomly and uniformly selected as regions of interest (ROIs). The number of nuclei present in each ROI was between 195 and 205 as counted by SYTO13 staining. Whole transcriptomic sequencing was performed on 47 ROIs per slide.

### Sequencing and RNA data processing

After selecting ROIs, the bound RNA probes were photocleaved and individual segmented areas were collected for sequencing before amplifying Illumina sequencing libraries. AMPure XP beads (Beckman Coulter, A63880) were used to purify PCR reactions at a 1.2× bead-to-sample ratio. DNA high sensitivity bioanalyzer assay (Agilent Technologies) and Qubit dsDNA high sensitivity assay (Thermo Fisher Scientific) were used to evaluate the size and concentration of the sequencing library. Total target counts were calculated from the total sampled areas (μm^2^) and the sequencing depths were 100 counts/μm^2^. All sequencing libraries were generated with index uniquely, which were allowed to be pooled together for sequencing. After loaded at a concentration of 365 pM with 5% PhiX, WTA libraries were sequenced with an Illumina NovaSeq 6000 platform under the following parameters: read 1, 27 cycles; read 2, 27 cycles; index 1, 8 cycles; index 2, 8 cycles. GeoMx NGS Pipeline was used to process RNA data. The reads were trimmed, merged, and aligned to indexing oligos. Before converting digital counts from reads, the PCR duplicate reads were removed according to the unique molecular identifier region. Targets that fell below the limit of quantitation were removed, which was defined as the geometric mean + 2 SD of the negative control probes, and the upper quartile normalized dataset was used.

### DEGs and enrichment analysis

DEGs of GeoMx RNA data between two groups were determined by unpaired two-tailed student’s t test with fold change >2 or <0.5, and *p* < 0.05 was considered statistically significant. The multiple test correction of Benjamini–Hochberg was used to adjust the *p*-values of individual genes. The volcano plots of DEGs were visualized by Hiplot (https://hiplot.org)^59^. The GO Biological Process enrichment of DEGs were analyzed and visualized using ClusterProfiler R package^60^. The network of pathways was visualized by ShinyGO^61^.

### Deconvolution of cell abundance

Cell counts and proportions were calculated using the SpatialDecon R package^62^ based on the cell profile matrix from the Mouse Cell Atlas adult liver dataset^63^. Heatmaps of NPCs abundance were visualized by TBTools^64^. Each column represents a sample of the indicated zone in corresponding figures. We converted the Cibersort-derived leukocyte marker genes in LM22 to their mouse orthologous before deconvoluting the gene expression profiles of each ROI through Cibersort R package to assess the immune cells’ infiltration profile.

### CMap and hub genes analysis

By querying selected differentially expressed genes (DEGs) against the CMap database^16^, we obtained reference perturbagen signatures most similar (or dissimilar) to our interested gene signatures. The small molecule candidate was screened by Venn diagram intersection based on three different queried gene sets. The celastrol-regulated gene signatures in the HepG2 cell line from the CMap database were analyzed to obtain the top 100 genes, whose overexpression or knockdown produced gene expression profile changes similar or opposite to those regulated by celastrol treatment. After analyzing the protein interaction network of the selected top 200 genes by STRING database, the top 9 hub genes were analyzed through the Cytohubba plugin of Cytoscape (v3.9.1) and ranked according to Maximum Neighborhood Component scores or average transcriptional impact scores of celastrol on HepG2 cell line obtained from CMap database.

### In silico molecular docking of celastrol with HIF1α protein

The structure of celastrol and deferoxamine-mesylate was downloaded from the Traditional Chinese Medicine Systems Pharmacology Database and Analysis Platform^65^ and ZINC database (https://zinc.docking.org/). The structures of HIF1α protein (PDB ID 4ZPR and 4H6J) were downloaded from Protein Data Bank^66^. In silico molecular docking was performed by cavity-detection guided blind docking^67^.

### Cell culture and luciferase assay

Human embryonic kidney (HEK) 293T cell lines were cultured in Dulbecco’s modified Eagle’s medium (DMEM; G4511, Servicebio, Wuhan, China) with 10% fetal bovine serum (FBS; 04-001-1ACS, Biological Industries, Israel) and 1% penicillin-streptomycin (G4003, Servicebio, Wuhan, China) in a 37 °C incubator with 5% CO_2_. HEK293T cells were transfected with HIF1α, ARNT, tk-HRE-luciferase, and Renilla luciferase plasmids for 24 h. The transfected cells were treated with 100 nM celastrol or 10 μM CoCl_2_ for 24 h before the dual-luciferase reporter gene analysis with a commercial kit (RG027; Beyotime, Beijing, China). CoCl_2_ was used as a positive control to activate HIF1α. The transfection efficiency was normalized against Renilla luciferase activity.

### Quantitative PCR analysis

Total RNA was extracted (abs9331; Absin, Shanghai, China) from mouse livers and cDNA was prepared by using FastKing RT Kit (With gDNase) (KR116; TIANGEN, Beijing, China). SYBR Green-based real-time PCR (abs601511; Absin, Shanghai, China) was performed with the Applied Biosystems QuantStudio 1 System. The relative gene expression levels were normalized against the Gapdh housekeeping gene. The qPCR primer sequences used are listed in Supplementary Table 1.

### Western blot analysis

Mouse livers were lysed with RIPA lysis buffer (G2002, Servicebio, Wuhan, China) containing a protease inhibitor (4693159001; Roche, Basel, BS, Switzerland) and phosphatase inhibitor (P1081; Beyotime, Beijing, China). Total protein was extracted by vortex and centrifugation at 4 °C and then quantified with a BCA kit (G2026; Servicebio, Wuhan, China). Samples containing equal quantities of protein were separated by 12% SDS-PAGE, transferred to PVDF membranes (IPVH00010; Millipore, Billerica, MA, USA), blocked with 5% skim milk in 0.5‰TBST for 1 h, incubated with the indicated antibodies (1:1000 for VEGFA and 1:10000 for GAPDH) overnight at 4 °C and incubated with the HRP-conjugated secondary antibodies (1:10000) for 1 h. Finally, protein bands were detected with an ECL kit (34580; Thermo Fisher Scientific, Waltham, MA, USA). The antibodies used are listed in Supplementary Table 2. Quantification of the band intensity was performed using ImageJ software.

### Statistics and reproducibility

All statistical analyses were performed with the GraphPad Prism software (v8.0.1). Data were expressed as mean ± SEM. Two independent groups’ comparisons were performed using the student’s t test and three or more groups’ comparisons were performed using one-way ANOVA or two-way ANOVA, with *p* < 0.05 considered statistically significant. Detailed sample size and statistical parameters can be found in Supplementary Data 1.

### Reporting summary

Further information on research design is available in the Nature Portfolio Reporting Summary linked to this article.

## Supplementary information

Supplementary Information

Description of Additional Supplementary Files

Supplementary Data 1

Reporting Summary

## Data Availability

The sequencing data and processed data have been deposited in GEO database with the accession codes GSE217936.

## Code availability

All code used in this study is open-sourced and referenced.
