# Supplementary material for: Spatial transcriptomics analysis of zone-dependent hepatic ischemia-reperfusion injury murine model
Source: Commun Biol. 2023 Feb 18;6:194. doi: 10.1038/s42003-023-04564-0 (PMC9938905; doi:10.1038/s42003-023-04564-0)
Supplement: Supplementary file 1 — Supplementary Information [file 42003_2023_4564_MOESM1_ESM.pdf]

Spatial Transcriptomics Analysis of Zone-Dependent Hepatic Ischemia-Reperfusion Injury Murine Model

Jiaqi Xin[1], Ting Yang[2], Xiaoyi Wu[1], Yingting Wu[2], Yi Liu[2], Xuan Liu[1], Mengxi Jiang[2, 3*], Wei Gao[1, 4*]

Affiliations:

[1] School of Traditional Chinese Medicine, Capital Medical University, Beijing 100069, China.

[2] Department of Pharmacology, School of Basic Medical Sciences, Capital Medical University, Beijing, 100069, China.

[3] Advanced Innovation Center for Human Brain Protection, Capital Medical University, Beijing, 100069, China.

[4] Beijing Shijitan Hospital, Capital Medical University, Beijing, 100038, China.

*** Corresponding authors:** Wei Gao. Email: weigao@ccmu.edu.cn; Mengxi Jiang. Email: jmx@ccmu.edu.cn.

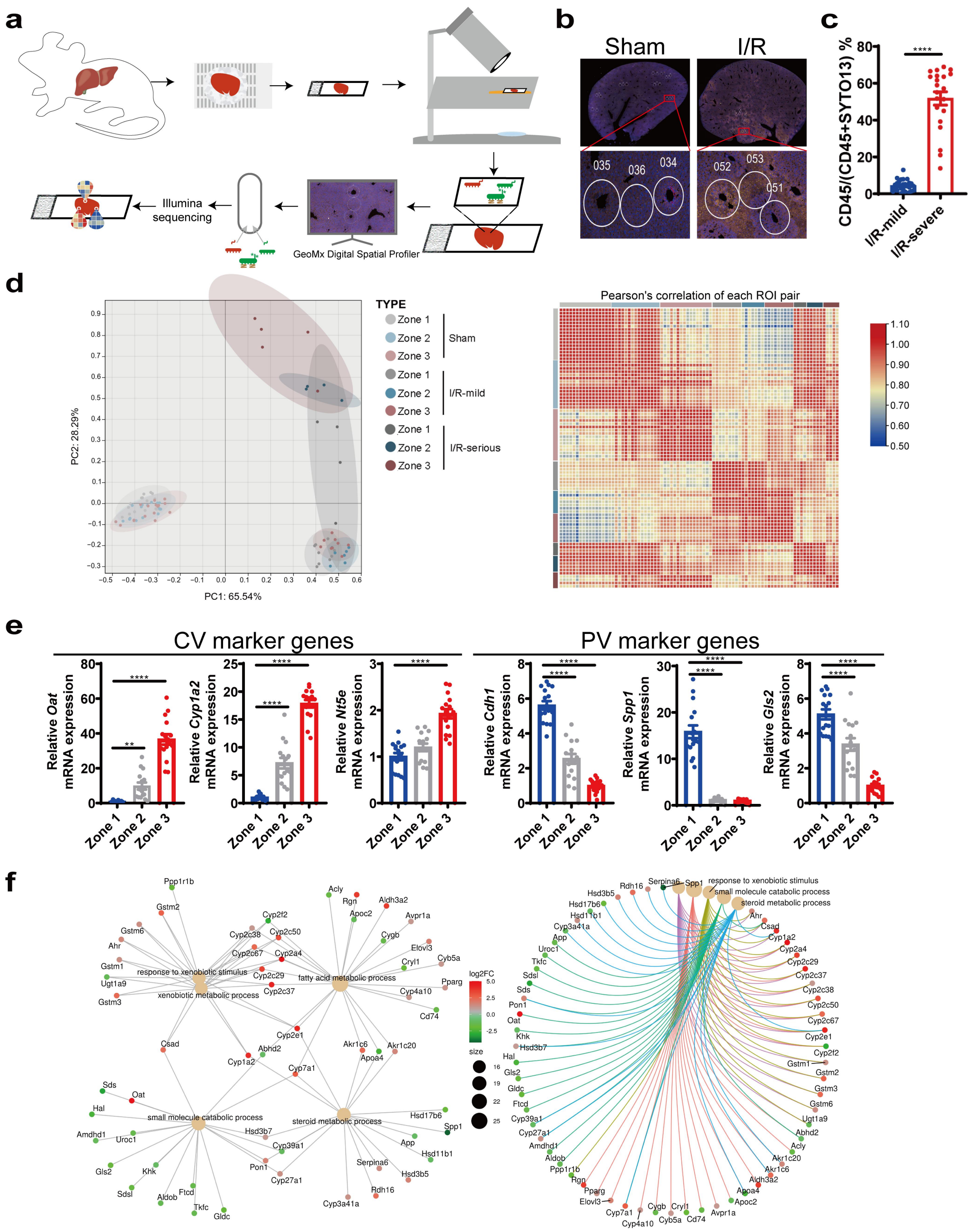

Supplementary Fig 1. Hepatic zone-specific functional pathways and KCs abundance at the steady state.

a Workflow of spatial transcriptomics. **b** Representative ROIs images of sham and I/R group. **c** CD45 positive rates calculated as CD45/(CD45+SYTO13) by ImageJ software (Student's t test, n=21-26, ****p < 0.0001). **d** PCA and ROI correlation heatmap of each ROI pair. **e** Expression of representative CV and PV marker genes (One-way ANOVA, n=15-16, **p < 0.01, ****p < 0.0001). **f**

Zone 3-specific top GO and DEGs network. The node color represents $\log_2$ fold change in gene expressions of zone 3 versus zone 1. Data were expressed as mean ± SEM.

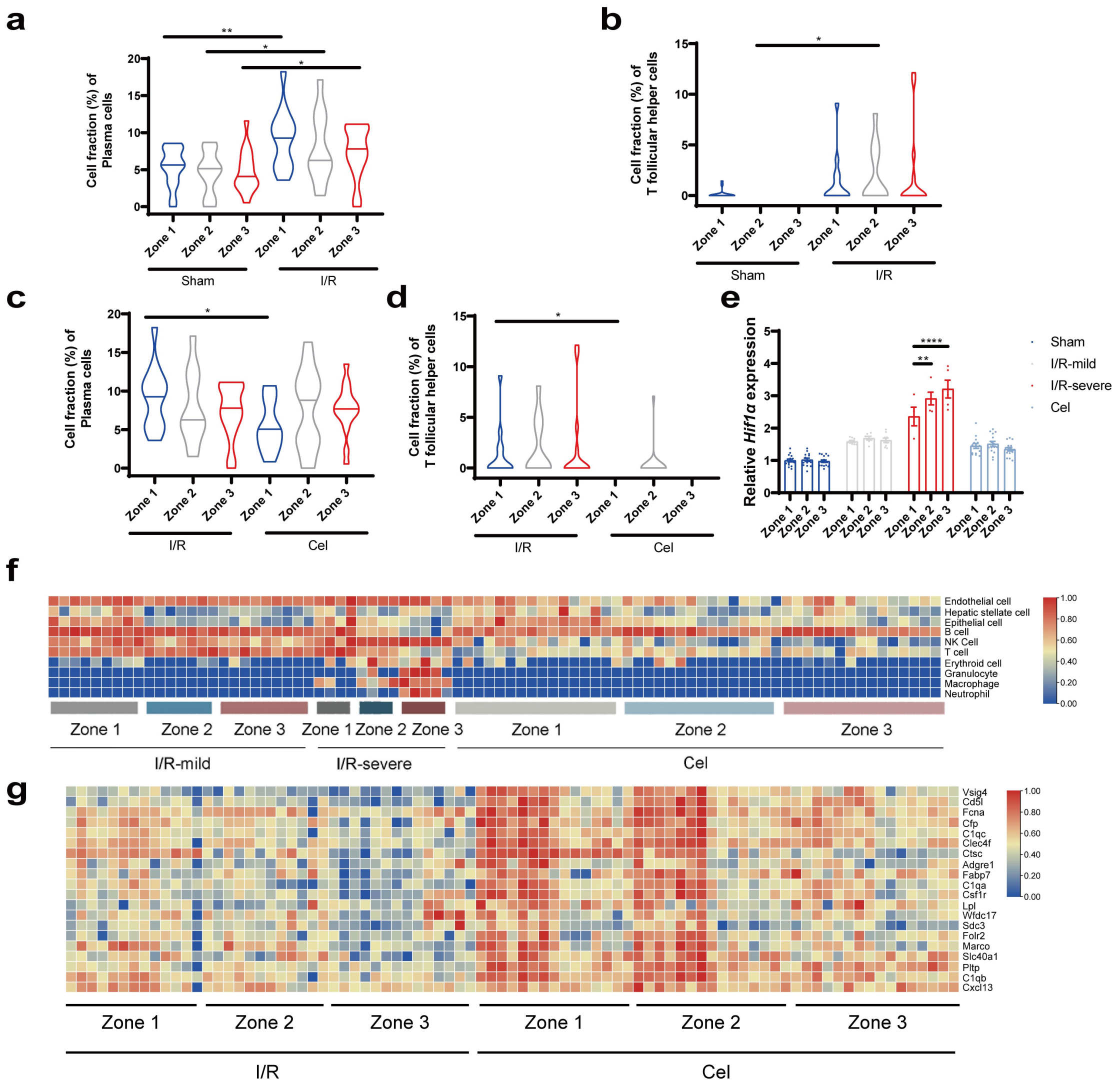

Supplementary Fig 2. The abundance of hepatic NPCs after I/R injury and celastrol treatment.

a-d Cell fractions of plasma cells and Tfhs were calculated by Cibersort algorithm (Center lines indicate median value. Student's t test, n=11-16, *$p < 0.05$, **$p < 0.01$). **e** Relative HIF1α expression in each zone of different groups (Two-way ANOVA, n=15-16 for Sham and Cel group, and n=7-9 for I/R mild regions and n=4-5 for I/R severe regions, **$p < 0.01$, ****$p < 0.0001$, data were expressed as mean ± SEM). **f** The scaled proportion of NPCs in I/R and celastrol group. **g** The scaled Kupffer cells marker genes expression in I/R and celastrol group.

59
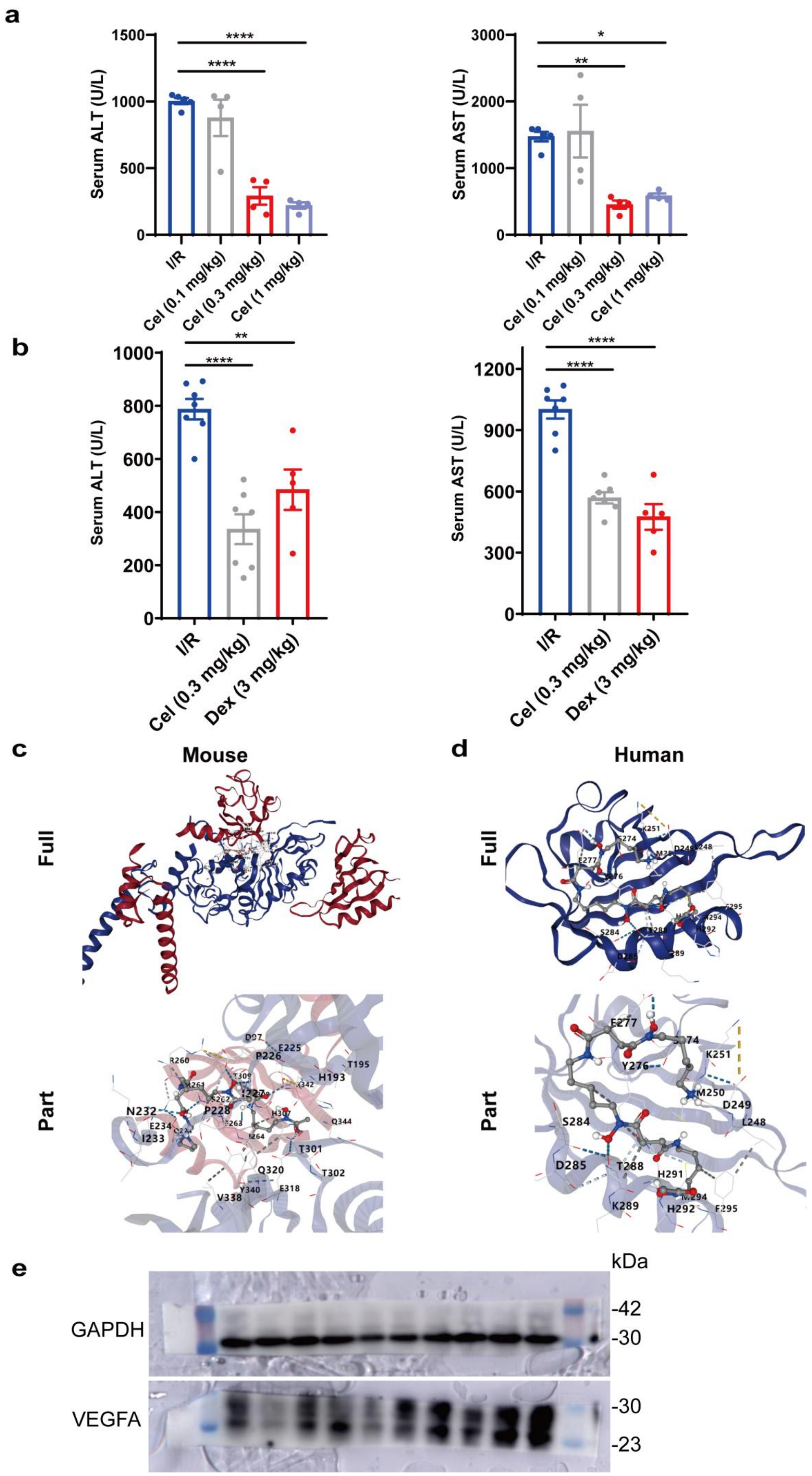
a
Serum ALT (U/L)
Serum AST (U/L)
I/R
Cel (0.1 mg/kg)
Cel (0.3 mg/kg)
Cel (1 mg/kg)
b
Dex (3 mg/kg)
c
Mouse
d
Human
Full
Part
e
kDa
GAPDH
VEGFA
-42
-30
-23

Supplementary Fig 3. Celastrol reduced I/R injury and activated the hypoxia pathway.

a, b Effect of one-week pre-treatment of celastrol or dexamethasone sodium phosphate on Serum transaminase levels after I/R injury (One-way ANOVA, n=4-7, $*p < 0.05$, $**p < 0.01$, $****p < 0.0001$, data were expressed as mean ± SEM). **c, d** *In silico* molecular docking results of deferoxamine-mesylate with mouse HIF1α protein in **c** and human HIF1α protein in **d**. **e** Unprocessed gels of Fig. 7h.

Supplementary Table 1: qPCR primer sequences

Gene	Forward Primer (5’-3’)	Reverse Primer (5’-3’)
Il-6	TACCACTTCACAAGTCGGAGGC	CTGCAAGTGCATCATCGTTGTTC
Il-1β	TTGACGGACCCCAAAAGATG	AGGACAGCCCAGGTCAAAG
Mcp-1	ACTCACCTGCTGCTACTCATTCAC	GTATGTCTGGACCCATTCCTTCTT
Gapdh	AACTTTGGCATTGTGGAAGG	ACACATTGGGGGTAGGAACA
Vegf	GGCCTCCGAAACCATGAACT	CTGGGACCACTTGGCATGG
Hif1α	CCCATTCCTCATCCGTCAAATA	CCTGTGGTGACTTGTCCTTTAG

Supplementary Table 2: Antibody information

Antibody	Company	Cat No.
Anti-VEGF	Bioss	Bs-1313R
Anti-GAPDH	Proteintech	10494-1-AP
Anti-iNOS	Servicebio	GB11119
Anti-CD163	Servicebio	GB113751
Anti-F4/80	Servicebio	GB113373
Anti-Rabbit IgG	Proteintech	SA00001-2

Supplementary Table 3: Putative docking sites between celastrol/deferoxamine-mesylate and HIF1α

Ligand	Protein	Binding Energy (kcal/mol)	Cavity Size (Å^3)	Center			Size		
				x	y	z	x	y	z
Celastrol	Mouse	-9.6	512	-124	-49	6	23	23	23
	HIF1α	-9.4	548	-112	-61	-31	23	23	23
		-8.8	3811	-114	-44	1	23	23	23
		-8.3	497	-106	-58	-15	23	23	23
		-7.4	1595	-95	-34	14	23	23	23
	Human	-7.3	44	19	-17	-35	23	23	23
	HIF1α	-6.7	33	12	-13	-21	23	23	23
		-6.6	30	16	7	-28	23	23	23
		-6.4	31	16	1	-17	23	23	23
		-6.1	36	24	3	-23	23	23	23
Deferoxamine-mesylate	Mouse	-6.6	3811	-114	-44	1	38	38	38
	HIF1α	-6.4	512	-124	-49	6	38	38	38
		-6.3	1595	-95	-34	14	38	38	38
		-6	548	-112	-61	-31	38	38	38
		-5.5	497	-106	-58	-15	38	38	38
	Human	-4.7	30	16	7	-28	38	38	38
	HIF1α	-4.6	33	12	-13	-21	38	38	38
		-4.5	44	19	-17	-35	38	38	38
		-4.3	36	24	3	-23	38	38	38
		-4.3	31	16	1	-17	38	38	38
