# Supplementary material for: Spatial transcriptomics analysis of zone-dependent hepatic ischemia-reperfusion injury murine model
Source: Commun Biol. 2023 Feb 18;6:194. doi: 10.1038/s42003-023-04564-0 (PMC9938905; doi:10.1038/s42003-023-04564-0)
Supplement: Supplementary file 2 — Description of Additional Supplementary Files [file 42003_2023_4564_MOESM2_ESM.pdf]

Description of Additional Supplementary Files

File name: Supplementary Data 1
Description: The source data of the main figures.
